# Adaptation, personalization and capacity in mental health treatments: a balancing act?

**DOI:** 10.1097/YCO.0000000000000834

**Published:** 2022-10-22

**Authors:** Sophie D. Bennett, Roz Shafran

**Affiliations:** UCL Great Ormond Street Institute of Child Health, London, UK

**Keywords:** adaptation, implementation, mental health, personalization

## Abstract

There are increasing calls for mental health treatments to be adapted for different groups to maximize their acceptability and benefit to patients. However, adaptations can be costly to develop and evaluate, difficult to implement in routine clinical practice and may reduce service capacity at a time when there is unprecedented unmet need. An alternative method is personalization on an individual level. This review provides an overview of the issues related to personalization and adaptation of mental health interventions.

**Recent findings:**

Several terms have been used to describe changes to existing therapies, these reflect different extents to which existing treatments have been changed. Evidence-based practice and modular therapies allow a level of flexibility within intervention delivery without formal changes and not all changes to therapy should be considered as a new/adapted treatment but instead regarded as ‘metacompetence’. Implementing existing interventions in new contexts is preferable to developing new interventions in many instances. New guidance outlines how researchers can adapt and transfer interventions to varied contexts.

**Summary:**

The review provides proposed definitions of different changes to therapy. Modified and personalized treatments may improve acceptability to patients whilst maximizing implementation of evidence-based practice within clinical services.

## INTRODUCTION

Estimates suggest that in 2019, one in eight people around the world were living with a mental health disorder [1]. Demand for services is increasing, and there is simply insufficient capacity to manage this within existing provision [2]. Many people are not able to access evidence-based support for mental health difficulties [3,4]. At the same time, there are increasing calls for mental health treatments to be tailored to maximize their acceptability and benefit to patients [5]. Although evidence-based psychological interventions can be highly efficacious for treating common mental health difficulties such as anxiety [6], effect sizes mask individual variation as not all patients benefit from these interventions [7]. However, creating new or ‘adapted’ treatments for different client groups is costly, requires new therapist training, and requires that therapists in services are trained in multiple interventions [8,9], which may further reduce service capacity as fewer therapists would be competent to deliver the intervention. How can we balance these needs of access and personalization when developing new interventions in mental health research? This review provides an overview of the issues related to adaptation, personalization and implementation within mental health and innovate methods of addressing this tension, such as modular treatments or computerized treatments based on measurement of mental health symptoms in individuals. 

**Box 1:**
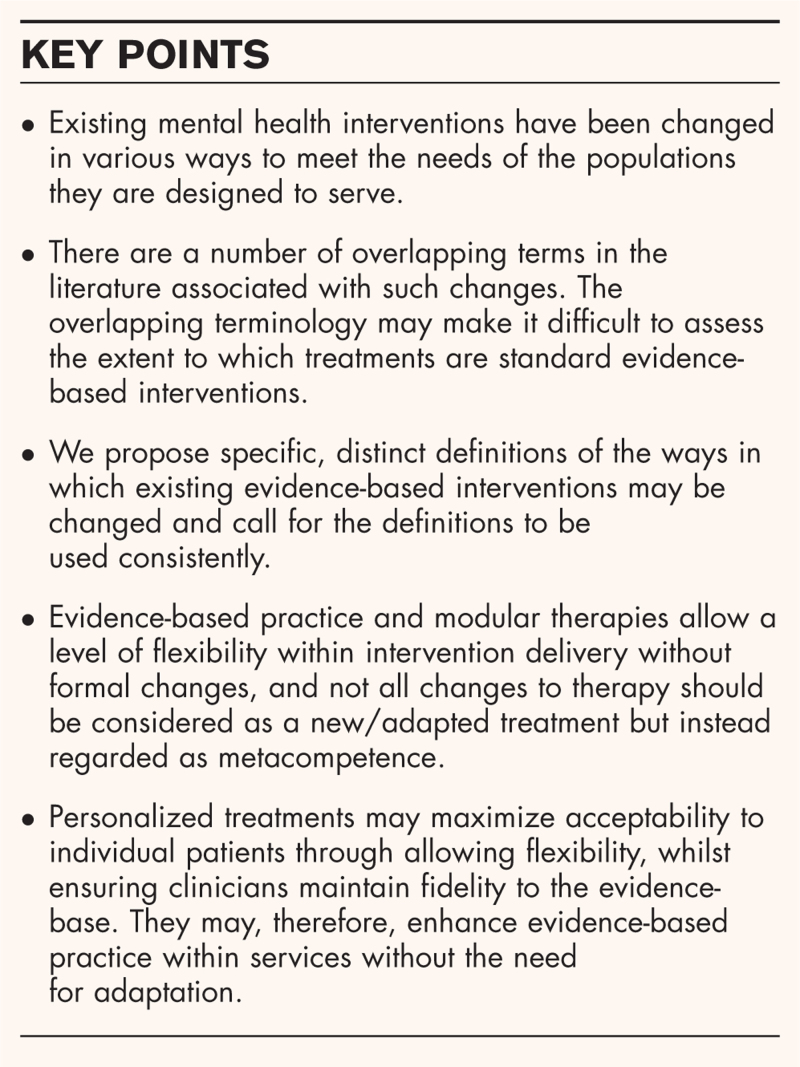
no caption available

## DEFINITIONS

**FIGURE 1:**
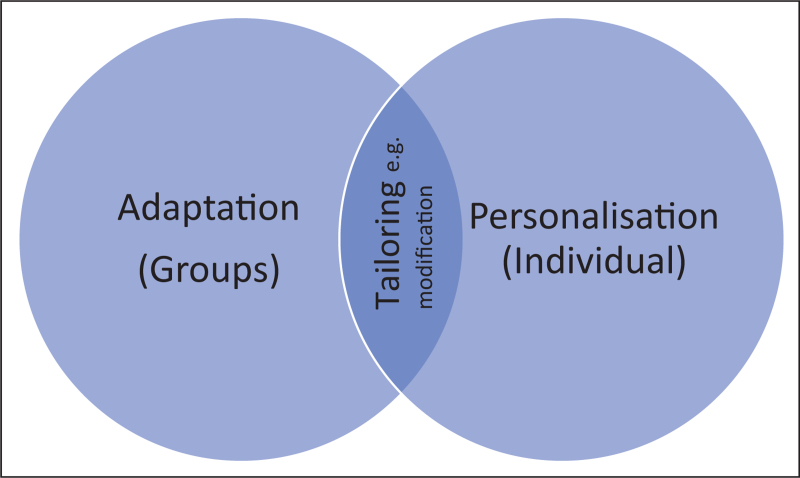
Conceptual diagram illustrating how terms are linked.

There are a number of overlapping terms in the literature associated with changes to evidence-based interventions. The overlapping terminology may make it difficult to assess the extent to which treatments are ‘standard’ evidence-based interventions, and therefore, how confident services are to implement them without further training or specialist staff. Dictionary definitions of personalization (‘to make something suitable for the needs of a particular person’) and adaptation (‘the action or process of changing something, or of being changed, to suit a new purpose or situation’) demonstrate that these concepts are related, yet different. Personalization suggests that an individual treatment can be changed to suit an *individual* client, whereas adaptation suggests that treatments are changed to suit *groups* of clients. ‘Tailoring’ (‘make or adapt for a particular purpose or person’), can refer to both groups or individuals. Table 1 outlines the different terms that have been used to describe changes to therapy protocols in the literature and Fig. 1 demonstrates how these concepts link together.

**Table 1:** Different terms that have been used to describe changes to therapy protocols in the literature

Concept	Dictionary definition	What this means for mental health interventions
Adaptation	The action or process of changing something, or of being changed, to suit a new purpose or situation	Changing an intervention for a group of people. However, the ways in which the intervention is changed can be hugely varied, ranging from changing the core content of the intervention, to amending language or examples to increase their relevance to different populations, to ‘organization specific’ adaptations [6], such as changing the location of services or time/length of intervention, or mode of delivery (e.g. face-to-face to app).
Personalization	To make something suitable for the needs of a particular person	Personalized treatments are those than can be changed to suit individual patients, for example, modular treatments or treatments based on providing different strategies according to measurements and/or personal preferences.
Tailoring	Make or adapt for a particular purpose or person	Can refer to both groups or individuals – covers both adaptation and personalization.
Modification	Something that is changed slightly, especially to improve it or make it more acceptable or less extreme	A modified treatment suggests that the original treatment forms the vast majority of the new treatment, but there have been slight changes to content, for example, using examples or language related to specific client groups to improve its acceptability or effectiveness.
Expansion	The increase of something in size, number or importance	Suggests that the original treatment remains but that material has been added.

Treatments can be adapted or personalized in different ways. A recent review of adaptations to cognitive behavioural therapy (CBT) for adolescents with comorbid depression and chronic illness found that adaptations included those to both content and delivery [10^▪▪^]. Content adaptations included cognitive restructuring of illness-related thoughts, behavioural activation balancing illness-related and enjoyable activities, and psychoeducation of the link between chronic illness and mental health. Adaptations to the delivery included being flexible through the use of the telephone and including parents in the intervention. A systematic review and conceptual typology of adapted psychological interventions for people from ethnic minority groups [11^▪▪^] categorized adaptations that were therapist-related, content-related and organization-specific (e.g. location of services, pathways into care, time or length of intervention). There was evidence for greater efficacy for adapted interventions compared with nonadapted treatments, with suggestion that organization-specific adaptations may have the greatest benefits.

## ADAPTATION VERSUS IMPLEMENTATION

Cuijpers *et al.*[12] argues that the focus on randomized controlled trial evidence of adapted versions of interventions for depression may be unhelpful, ‘because the evidence indicates that all types and formats with human involvement are effective in all specific target groups’. Adapting interventions to suit different populations may potentially increase their efficacy, although that has yet to be established, but there are significant costs of developing and trialling an intervention and training therapists to deliver it [9]. There may also be costs associated with confidence of therapists to deliver interventions within routine practice and for therapists to be able to learn multiple different interventions for their client groups [9]. In the case of chronic illness, for example, many clinicians feel ill-equipped to manage mental health difficulties in this context [13]; there is evidence that standard, un-adapted interventions are effective [14] but not implemented routinely [15]. Arguably, focusing on implementing standard, un-adapted interventions would help meet a huge demand for mental health support in this population as large numbers of clinicians are already trained in these interventions [16]; the creation of specially adapted interventions would imply that standard interventions should not be used; therefore, further disadvantaging this already disadvantaged group of patients.

However, while un-adapted interventions are effective, and we may not need to create new, specialist treatments for each patient group, or assess the efficacy of old treatments in new populations, there is good reason to adapt interventions to new situations. There is a known implementation gap for evidence-based practice in CBT [17–20]. Many people still do not access evidence-based interventions [19], and there are many barriers to the dissemination of evidence-based psychological treatments [20]. Such gaps may be addressed through implementation science.

Moore *et al*. [21^▪▪^] have developed the ‘ADAPT guidance’, recognizing that ‘Implementing interventions with a previous evidence base in new contexts might be more efficient than developing new interventions for each context’ but that ‘although some interventions transfer well, effectiveness and implementation often depend on the context’. The guidance outlines methods to adapt existing interventions for new *contexts*, including new settings and healthcare systems or new populations or population subgroups. They suggest that this may support efforts to reduce inequalities through ensuring interventions are appropriate for the needs of disadvantaged groups. Their approach emphasizes the importance of implementation science rather than creation of new interventions. Implementation Science is: ‘the scientific study of methods and strategies that facilitate the uptake of evidence-based practice and research into regular use by practitioners and policymakers’ and implementation-focused trials that centre on such factors are likely to be of value.

## HOW CAN INTERVENTIONS BE ADAPTED TO MAXIMIZE IMPLEMENTATION?

There are two key points to be kept in mind when adapting interventions to maximize implementation:

### Evidence-based practice and ‘good’ therapy

The new Medical Research Council (MRC) and National Institute for Health Research (NIHR) complex intervention research framework provides a structure for developing and evaluating complex interventions [22]. It states that within development of a complex intervention, the complexity may relate to the ‘level of flexibility or tailoring of the intervention or its components that is permitted (i.e. how dynamic or adaptive the intervention is)’ and that ‘flexibility in intervention delivery and adherence might be permitted to allow for variation in how, where, and by whom interventions are delivered and received. Standardization of interventions could relate more to the underlying process and functions of the intervention than on the specific form of components delivered’. They provide the example of surgical trials in which the protocol can be designed with flexibility for intervention delivery; there is no suggestion that such flexibly delivered interventions are different from each other. This suggests that interventions do not need to be ‘adapted’ in order to flexibly meet the needs of specific patients or patient groups.

This concept has also been described by Kendall *et al.*[23^▪^] as ‘flexibility within fidelity’. Kendall *et al.* have described how therapists are prone to cognitive processing errors, assuming that the specific circumstances of the client or client groups we work with make them different to the empirical findings of trials, and therefore, discount standard evidence-based interventions. In reality, ‘actuarial prediction is superior to clinical prediction’ (p. 2), and therefore, it is likely to be more beneficial to use the evidence-based treatment. Kendall *et al.* suggest that rather than using a nonevidence-based treatment, therapists should ‘take the client's specifics into account and apply the known-to-be-effective treatment with flexibility’ (p. 2).

This recommended flexibility is fundamental to CBT, which is recommended by guidelines as a first-line treatment for many common mental health problems globally. Competence frameworks for CBT therapists, which describe the skills, competent CBT therapists should possess [24], state a number of ‘metacompetencies’ in line with this principle:

(1) Capacity to implement treatment models in a flexible but coherent manner.
(2) Capacity to adapt interventions in response to client feedback.
(3) Capacity to use and respond to humour.
(4) Capacity to implement CBT in a manner consonant with its underlying philosophy.
(5) Capacity to formulate and to apply CBT models to the individual client.
(6) Capacity to select and skilfully to apply the most appropriate CBT intervention method.
(7) Capacity to structure sessions and maintain appropriate pacing.
(8) Capacity to manage obstacles to carrying out CBT.

Evidence-based interventions such as CBT are inherently flexible, should be personalized to meet the needs of individual clients and therapists who do not modify the treatment are not competent therapists delivering good therapy. This is consistent with evidence-based practice within medicine more broadly. The definition of evidence-based practice constitutes three components: research evidence, clinical expertise and patient values, preferences and characteristics [25], that is, that interventions are not based on the research evidence alone, but that the research evidence is applied flexibly, accounting not only for the clinician's own expertise but also the patient's specific needs.

### Personalization

Whilst a multitude of specific, adapted interventions tested in randomized controlled trials may not be a feasible way to meet the current mental health needs of the population, personalized interventions may be a helpful way forward. Changing the individual treatment to suit individual clients in this way allows for one standardized intervention to be applied flexibly across a range of populations or presenting difficulties. There is some evidence that patients prefer personalized interventions, and that personalization may enhance adherence and engagement [26].

One method by which personalization can be achieved is modular treatments. Modular treatments, in which therapists and/or clients can select the elements of protocols most suited to their presentation and needs allow protocolized, standard interventions to be delivered flexibly, allowing for patient variation. Decisions regarding which modules or elements are used may be based on clinical judgement, patient choice, data from outcome measurement or a combination [27]. For example, the Modular Approach to Therapy for Children with Anxiety, Depression, Trauma or Conduct problems (MATCH ADTC; [28]) combined modules for four common mental health problems for children and young people, allowing treatment of more than one area of difficulty. This accounts for the high rates of co-occurring mental health difficulties in children and young people seen in clinical practice. A therapist ‘dashboard’ containing scores from weekly measurements of goal progress and symptoms together with a flow-chart/algorithm guides module choice. The MATCH-ADTC intervention is inherently personalized but has also been modified to suit different contexts. For example, in line with the ADAPT guidance, one study modified and expanded the intervention to enable the intervention to be implemented within the context of physical healthcare services [29]. Rather than developing a new intervention from scratch, implementation science methods, including a systematic literature review, focus groups of families and clinicians, plan-do-study-act cycles and qualitative interviews, were used to modify and expand the existing intervention to meet the needs of this different setting. Overall, the content of the intervention remained largely unchanged but additional modules were added specific to the needs of families and young people with epilepsy, and brief training was developed to support delivery from within a physical healthcare setting. Normalization Process Theory was used to guide this process to ensure that the intervention would be sustained in routine practice.

Other ways in which interventions may be personalized include maximizing the use of technology, for example, using apps to collect measures of mental health symptoms and providing different strategies according to the results of those measures (see [30]). It is also possible for digital interventions to be personalized through allowing patients to choose the content they consider most relevant to them [31].

## CONCLUSION

A number of terms have been used to describe changes to existing therapies, including adaptation, personalization, modification, tailoring and expansion. These reflect different extents to which existing treatments have been changed. It is recommended that that these terms are used consistently, and that the flexibility inherent in evidence-based practice means that standard protocols can be used across a variety of mental health patients and settings without the need for extensive revision. When needed, ADAPT guidance outlines how researchers can adapt and transfer interventions to new contexts. Modified and personalized treatments may balance the need for personalization with the implementation of standardized interventions, hence improving relevance and acceptability to patients whilst maximizing implementation within clinical services.

## Acknowledgements

*We would like to thank Professor Isobel Heyman for insightful discussions regarding adapting therapies for children and young people with additional complexities, such as those with chronic physical illnesses.*

*All research at Great Ormond Street Hospital NHS Foundation Trust and UCL Great Ormond Street Institute of Child Health is made possible by the NIHR Great Ormond Street Hospital Biomedical Research Centre. The views expressed are those of the author(s) and not necessarily those of the NHS, the NIHR or the Department of Health.*

### Financial support and sponsorship

*None.*

### Conflicts of interest

*There are no conflicts of interest.*

## REFERENCES AND RECOMMENDED READING

Papers of particular interest, published within the annual period of review, have been highlighted as:

▪ of special interest
▪▪ of outstanding interest

## References

[1] Institute of Health Metrics and Evaluation. Global Health Data Exchange (GHDx) 2022. Available at: https://vizhub.healthdata.org/gbd-results/. [Accessed 9 October 2022].

[2] Baker C. Mental health statistics (England) 2021. Available at: https://researchbriefings.files.parliament.uk/documents/SN06988/SN06988.pdf. [Accessed 9 October 2022].

[3] Baker C. Mental health statistics for England: prevalence, services and funding 2020. Available at: https://dera.ioe.ac.uk/34934/1/SN06988%20(redacted).pdf. [Accessed 9 October 2022].

[4] Kilbourne AM Beck K Spaeth-Rublee B. Measuring and improving the quality of mental healthcare: a global perspective. World Psychiatry 2018; 17:30–38. 29352529 10.1002/wps.20482 PMC5775149

[5] Ng MY Weisz JR. Annual research review: building a science of personalized intervention for youth mental health. J Child Psychol Psychiatry 2016; 57:216–236. 26467325 10.1111/jcpp.12470 PMC4760855

[6] Cuijpers P Cristea IA Karyotaki E. How effective are cognitive behavior therapies for major depression and anxiety disorders? A meta-analytic update of the evidence. World Psychiatry 2016; 15:245–258. 27717254 10.1002/wps.20346 PMC5032489

[7] Boschloo L Bekhuis E Weitz ES. The symptom-specific efficacy of antidepressant medication vs. cognitive behavioral therapy in the treatment of depression: results from an individual patient data meta-analysis. World Psychiatry 2019; 18:183–191. 31059603 10.1002/wps.20630 PMC6502416

[8] Herschell AD Kolko DJ Baumann BL Davis AC. The role of therapist training in the implementation of psychosocial treatments: a review and critique with recommendations. Clin Psychol Rev 2010; 30:448–466. 20304542 10.1016/j.cpr.2010.02.005 PMC2872187

[9] Crome E Shaw J Baillie A. Costs and returns on training investment for empirically supported psychological interventions. Aust Health Rev 2017; 41:82–88. 27007500 10.1071/AH15129

[10▪▪] Morey A Loades ME. Review: how has cognitive behaviour therapy been adapted for adolescents with comorbid depression and chronic illness? A scoping review. Child Adolesc Ment Health 2021; 26:252–264. 32951336 10.1111/camh.12421

[11▪▪] Arundell L-L Barnett P Buckman JEJ. The effectiveness of adapted psychological interventions for people from ethnic minority groups: a systematic review and conceptual typology. Clin Psychol Rev 2021; 88:102063. 34265501 10.1016/j.cpr.2021.102063 PMC8591374

[12] Cuijpers P. Four decades of outcome research on psychotherapies for adult depression: an overview of a series of meta-analyses. Can Psychol 2017; 58:7–19.

[13] Carroll S Moss-Morris R Hulme K Hudson J. Therapists’ perceptions of barriers and facilitators to uptake and engagement with therapy in long-term conditions. Br J Health Psychol 2021; 26:307–324. 33043530 10.1111/bjhp.12475

[14] Bennett S Shafran R Coughtrey A. Psychological interventions for mental health disorders in children with chronic physical illness: a systematic review. Arch Dis Child 2015; 100:308–316. 25784736 10.1136/archdischild-2014-307474

[15] Welch A Shafran R Heyman I. Usual care for mental health problems in children with epilepsy: a cohort study. F1000Res 2018; 7:1907. 32494354 10.12688/f1000research.15492.1 PMC7233177

[16] Ludlow C Hurn R Lansdell S. A Current Review of the Children and Young People's Improving Access to Psychological Therapies (CYP IAPT) Program: perspectives on developing an accessible workforce. Adolesc Health Med Ther 2020; 11:21–28. 32104131 10.2147/AHMT.S196492 PMC7023850

[17] Campion J Javed A Sartorius N Marmot M. Addressing the public mental health challenge of COVID-19. Lancet Psychiatry 2020; 7:657–659. 32531299 10.1016/S2215-0366(20)30240-6 PMC7282758

[18] Reardon T Harvey K Creswell C. Seeking and accessing professional support for child anxiety in a community sample. Eur Child Adolesc Psychiatry 2020; 29:649–664. 31410579 10.1007/s00787-019-01388-4 PMC7250799

[19] Harvey AG Gumport NB. Evidence-based psychological treatments for mental disorders: modifiable barriers to access and possible solutions. Behav Res Ther 2015; 68:1–12. 25768982 10.1016/j.brat.2015.02.004 PMC4395546

[20] Shafran R Clark DM Fairburn CG. Mind the gap: Improving the dissemination of CBT. Behav Res Ther 2009; 47:902–909. 19664756 10.1016/j.brat.2009.07.003

[21▪▪] Moore G Campbell M Copeland L. Adapting interventions to new contexts—the ADAPT guidance. BMJ 2021; 374:n1679. 34344699 10.1136/bmj.n1679 PMC8329746

[22] Skivington K Matthews L Simpson SA. A new framework for developing and evaluating complex interventions: update of Medical Research Council guidance. BMJ 2021; 374:n2061. 34593508 10.1136/bmj.n2061 PMC8482308

[23▪] Kendall PC. Flexibility within fidelity: breathing life into a psychological treatment manual. 2021; Oxford: Oxford University Press,

[24] Roth AD. Are competence frameworks fit for practice? Examining the validity of competence frameworks for CBT, psychodynamic, and humanistic therapies. Psychother Res 2015; 25:460–472. 24735091 10.1080/10503307.2014.906763

[25] Sackett DL. Evidence-based medicine. Semin Perinatol 1997; 21:3–5. 9190027 10.1016/s0146-0005(97)80013-4

[26] Cheek C Fleming T Lucassen MF. Integrating health behavior theory and design elements in serious games. JMIR Ment Health 2015; 2:e11. 26543916 10.2196/mental.4133 PMC4607397

[27] Schaeuffele C Schulz A Knaevelsrud C. CBT at the crossroads: the rise of transdiagnostic treatments. Int J Cogn Ther 2021; 14:86–113.

[28] Chorpita BF, Weisz JR. MATCH-ADTC: Modular approach to therapy for children with anxiety, depression, trauma, or conduct problems PracticeWise; 2009. https://www.amazon.co.uk/MATCH-ADTC-Approach-Children-Depression-Problems/dp/0984311513?asin=0984311513&revisionId=&format=4&depth=1

[29] Shafran R Bennett S Coughtrey A. Optimising evidence-based psychological treatment for the mental health needs of children with epilepsy: principles and methods. Clin Child Fam Psychol Rev 2020; 23:284–295. 31965422 10.1007/s10567-019-00310-3 PMC7192863

[30] Hollis C Falconer CJ Martin JL. Annual research review: digital health interventions for children and young people with mental health problems - a systematic and meta-review. J Child Psychol Psychiatry 2017; 58:474–503. 27943285 10.1111/jcpp.12663

[31] Andersson G Estling F Jakobsson E. Can the patient decide which modules to endorse? An open trial of tailored internet treatment of anxiety disorders. Cogn Behav Ther 2011; 40:57–64. 21337215 10.1080/16506073.2010.529457

